# Boys are more likely to be undernourished than girls: a systematic review and meta-analysis of sex differences in undernutrition

**DOI:** 10.1136/bmjgh-2020-004030

**Published:** 2020-12-15

**Authors:** Susan Thurstans, Charles Opondo, Andrew Seal, Jonathan Wells, Tanya Khara, Carmel Dolan, André Briend, Mark Myatt, Michel Garenne, Rebecca Sear, Marko Kerac

**Affiliations:** 1 Department of Population Health, London School of Hygiene and Tropical Medicine, London, UK; 2 Department of Medical Statistics, Faculty of Epidemiology and Population Health, London School of Hygiene and Tropical Medicine, London, UK; 3 National Perinatal Epidemiology Unit, Nuffield Department of Population Health, University of Oxford, Oxford, UK; 4 Institute for Global Health, University College London, London, UK; 5 Great Ormond Street Institute of Child Health, University College London, London, UK; 6 Emergency Nutrition Network, Kidlington, Oxfordshire, UK; 7 Department of Nutrition, Exercise and Sports, University of Copenhagen, Kobenhavn, Denmark; 8 School of Medicine, Center for Child Health Research,; 9 Brixton Health, Llwyngwril, Gwynedd, UK; 10 Institut de Recherche pour le Développement, UMI Résiliences, Bondy, France; 11 Institut Pasteur, Epidémiologie des Maladies Emergentes, Paris, France; 12 FERDI, Université d'Auvergne, Clermont‐Ferrand, France; 13 MRC/Wits Rural Public Health and Health Transitions Research Unit, School of Public Health, Faculty of Health Sciences, University of the Witwatersrand, Johannesburg, South Africa; 14 Centre for MARCH (Maternal, Adolescent & Reproductive Child Health), London School of Hygiene and Tropical Medicine, London, UK

**Keywords:** nutrition, systematic review, child health, public health

## Abstract

**Background:**

Excess male morbidity and mortality is well recognised in neonatal medicine and infant health. In contrast, within global nutrition, it is commonly assumed that girls are more at risk of experiencing undernutrition. We aimed to explore evidence for any male/female differences in child undernutrition using anthropometric case definitions and the reasons for differences observed.

**Methods:**

We searched: Medline, Embase, Global health, Popline and Cochrane databases with no time limits applied. Eligible studies focused on children aged 0–59 months affected by undernutrition where sex was reported. In the meta-analysis, undernutrition-specific estimates were examined separately for wasting, stunting and underweight using a random-effects model.

**Results:**

74 studies were identified: 44/74 studies were included in the meta-analysis. In 20 which examined wasting, boys had higher odds of being wasted than girls (pooled OR 1.26, 95% CI 1.13 to 1.40). 38 examined stunting: boys had higher odds of stunting than girls (pooled OR 1.29 95% CI 1.22 to 1.37). 23 explored underweight: boys had higher odds of being underweight than girls (pooled OR 1.14, 95% CI 1.02 to 1.26). There was some limited evidence that the female advantage, indicated by a lower risk of stunting and underweight, was weaker in South Asia than other parts of the world. 43/74 (58%) studies discussed possible reasons for boy/girl differences; 10/74 (14%) cited studies with similar findings with no further discussion; 21/74 (28%) had no sex difference discussion. 6/43 studies (14%) postulated biological causes, 21/43 (49%) social causes and 16/43 (37%) to a combination.

**Conclusion:**

Our review indicates that undernutrition in children under 5 is more likely to affect boys than girls, though the magnitude of these differences varies and is more pronounced in some contexts than others. Future research should further explore reasons for these differences and implications for nutrition policy and practice.

**Key questions**

**What is already known?**

- Undernutrition (wasting, stunting and underweight) is a public health problem affecting millions of children aged under 5 years globally.
- Although higher neonatal and infant morbidity/mortality for boys is well described, little attention has been given to sex differences in the field of undernutrition due to an assumption that girls are very often disadvantaged over boys.

**What are the new findings?**

- In most settings studied, undernutrition is more common among boys than girls, though the extent of these differences varies and is reversed in a few contexts.
- Both biological and social mechanisms have been proposed to be responsible for the observed differences as well as a combination of the two.

**Key questions**

**What do the new findings imply?**

- Greater awareness of actual sex differences is needed within the field of nutrition.
- While sex-specific data are routinely analysed and reported in nutrition surveys, it should be used in nutrition programming to better identify and understand what differences exist. Analysis should assess if the sex balance in programme admissions is reflective of the population undernutrition burden.
- Further research is needed to understand both the mechanisms behind and the reasons for that lead to sex and gender differences in undernutrition and their implications for nutrition policy and practice. Better epidemiological understanding is a priority, as is work to explore their consequent effects on morbidity and mortality.

## Introduction

Undernutrition is a serious public health problem affecting millions of children worldwide. Recent estimates indicate that stunting (low height-for-age) affects approximately 149 million children, with consequences for growth and cognitive development. Wasting (low weight-for-length), a life-threatening consequence of acute nutrient deficits and/or disease affects over 49 million children globally; 17 million of whom are severely wasted.^1^ However, these numbers are based on prevalence estimates meaning true numbers may be considerably higher when incidence is taken into consideration.^2^

Sex (referring to biological attributes) and gender (referring to socially constructed roles, behaviours and identities)^3^ are important considerations in the public health domain and receive different prominence in different academic and professional fields. Despite considerable research on childhood sex differences in neonatal and infant health, different disciplines often hold surprisingly contrary views on the relative vulnerability of male and female children.

In neonatal medicine and infant health communities, excess male morbidity and mortality is almost universally reported and is widely recognised.^4 5^ Boys are known to be more vulnerable than girls, from as early as the point of conception.^6^ Conditions common in childhood such as lower respiratory infections, diarrhoeal diseases, malaria and preterm birth are all more common in boys than girls, with the exception of measles, whooping cough and tuberculosis.^7^ All of these are not only causes of death but also of weight loss, growth faltering or severe undernutrition among young children.^8^ Boy–girl differences have not been explored in detail within the nutrition field, but girls are often widely viewed as more disadvantaged and vulnerable^9^ from a gender perspective.^10–13^

How underlying biological mechanisms related to sex and social differences in gender translate into the risk of anthropometric deficits and related morbidity and mortality in the field of nutrition remains understudied. Likewise, the practical implications of these differences remain to be determined. In terms of growth and nutrition status, sex differences have long been recognised and reflected through growth charts targeted at individual sexes.^14 15^ On average, boys are slightly heavier and longer at birth and throughout infancy compared with girls, and more detailed studies have shown that the extra average weight of boys is primarily lean mass, whereas fat mass is more similar between the sexes.^16 17^ To evaluate growth and nutritional status therefore, raw anthropometric data are conventionally converted to indices (eg, weight-for-age; weight-for-length, length-for-age) and expressed in comparison to reference populations as z-scores (SD scores, whereby +1 and −1 z-scores are one SD above and below the reference population median, respectively). Data published by WHO in 2006 represent a ‘gold standard’ of how children should grow and were developed from a globally representative reference population of healthy, breastfed children. In constructing the growth standards, data for boys and girls were analysed separately^15^ and the resulting growth charts should already therefore account for any sex differences. What has received little attention to date is whether sex differences reappear when z-scores are shifted away from the healthy reference range, which would indicate sex differences in susceptibility to undernutrition.

The objectives of this review were to systematically review the evidence for sex differences in undernutrition in children aged under 5 years, to explore evidence of any male/female differences in child undernutrition, and to document reasons given for any observed differences.

## Methods

This systematic review was conducted following the Preferred Reporting Items for Systematic reviews and Meta-Analyses (PRISMA) guidelines.^18^ A protocol for the review was defined, including inclusion and exclusion criteria, and was shared among authors for consensus. The protocol was then registered with the PROSPERO International prospective register of systematic reviews (CRD42018094818). The scope of this initial protocol was broad but as the review progressed and the heterogeneity of identified studies became increasingly apparent, we made a decision to divide our work into two parts: the first (this study) focuses on prevalence and recognition of sex-related differences; and the second, which will focus on the physiological and sociological mechanisms that may account for any identified differences.

### Search strategy

Our search strategy captured the concepts of undernutrition, sex and gender. Detailed search terms are in box 1.

Box 1 **Search terms**

1. undernutrition.mp. (5708)
2. malnutrition.mp. (39279)
3. malnutrition/ or exp fetal nutrition disorders/ or exp refeeding syndrome/ or exp severe acute malnutrition/ or exp kwashiorkor/ or exp starvation/ or exp wasting syndrome/ (25202)
4. (severe adj2 malnutrition).mp. (2131)
5. stunting.mp. (3456)
6. exp Growth Disorders/ (30538)
7. chronic malnutrition.mp. (519)
8. stunt*.mp. (6655)
9. MUAC.mp. (407)
10. mid upper arm circumference.mp. (771)
11. exp Nutritional Status/ (38539)
12. marasmus.mp. or Protein-Energy Malnutrition/ (7366)
13. famine.mp. (1726)
14. exp Starvation/ (9562)
15. (failure adj2 thrive).mp. (5307)
16. 1 or 2 or 3 or 4 or 5 or 6 or 7 or 8 or 9 or 10 or 11 or 12 or 13 or 14 or 15 (123406)
17. limit 16 to (“all infant (birth to 23 months)” or “newborn infant (birth to 1 month)” or “infant (1 to 23 months)” or “preschool child (2 to 5 years)”) (35919)
18. (boy* or girl* or male* or female* or gender or sex).ti, ab. (177252)
19. 17 and 18 (6631)

Numbers in parenthesis show the number of search results for each line.

Studies were identified by searching the Medline database using the above terms which were then adapted to Embase, Global health, Popline and Cochrane databases. No limits were applied for year of publication in order to capture historical publications on the subject. Studies were restricted to those that included terms for boy, girl, male, female, gender, or sex in the title or abstract, with the aim of filtering through the large body of literature that exists for undernutrition and capturing studies which either directly focused on sex and/or gender in the context of undernutrition or those which disaggregated and reported on it within main findings. As per the PRISMA recommendations, the search strategy was peer reviewed by a librarian.

### Eligibility criteria

Studies were included in the review if they met the following criteria: human studies, age range of 0–59 months, male and female participants, outcomes related to the prevalence or determinants of undernutrition, and related morbidity and mortality. Studies were eligible for inclusion in the meta-analysis if they presented data disaggregated by sex for both the overall sample and the outcome of interest (wasting, stunting, underweight), or relevant ORs. Studies of children over 59 months, non-English language, animal studies and studies on overweight/obesity and micronutrient deficiencies were excluded. Both peer-reviewed and grey literature were selected. In studies that included data for children both under and over 59 months, where possible, we extracted the data for children <59 months only. Where this was not possible, studies were excluded.

### Data extraction

All records identified through the search were imported into EndNote (EndNote V.X8, Clarivate Analytics). Duplicates were identified and removed. Initial screening was conducted by reading titles and abstracts to identify and remove studies which clearly did not fit our scope. Detailed review of the full text of all remaining results was conducted to determine which met the inclusion and exclusion criteria. When it was not clear how to classify an article, this was resolved through discussion and consensus with two or more authors.

A data extraction template was piloted on a small number of articles before being finalised. Data were extracted on study characteristics and outcomes of interest. These included aims and types of studies, sample size, prevalence and male/female ORs for undernutrition, and explanations offered for any differences identified.

### Analysis

Due to variations in type of paper and study design, the analysis was conducted in two parts: a qualitative systematic review followed by a meta-analysis. We performed random-effects meta-analyses to pool estimates from studies that included a measurement of undernutrition prevalence, or which assessed risks and determinants of undernutrition, and stratified results by sex. Missing counts, denominators and effect estimates such as ORs, relative risk and their associated CIs were calculated from other information provided where it was possible to do so. Studies that presented only adjusted ORs or risk ratios were excluded given that studies were likely to adjust for different factors and such adjusted effect estimates were not directly comparable. Undernutrition-specific estimates were pooled separately for wasting, stunting and underweight using a random-effects model. Analysis was also stratified by age and country. Pooled effects are presented as ORs and 95% CIs. Meta-regression was conducted to assess whether study-specific factors could explain the heterogeneity of effect estimates across studies. Statistical analysis was conducted using Stata V.15.1 (StataCorp 2017, Stata Statistical Software, College Station, Texas, USA).

In all studies conducted earlier than 2006, the National Center for Health Statistics (NCHS) growth^19^ references had been used. In all post-2006 studies that were included, the WHO (2006) growth standards for wasting, stunting and underweight, as measured through SD from the mean z-scores, were used. Wasting was defined by weight-for-height z-score <−2; stunting was defined by height-for-age z-score <−2; underweight was defined by weight-for-age z-score <−2.

### Risk of bias assessment

We adapted the National Heart, Lung and Blood institute study quality assessment tools for Observational cohort and cross-sectional studies to assess the quality of studies,^20^ and applied it to studies identified for the meta-analysis. Using this tool, we assessed data sources, a study’s presentation of aims and objectives and target populations, the appropriateness of anthropometric methods and the presentation of results. We adapted the tool to assess if studies acknowledged sex differences in the discussion of results.

### Patient and public involvement

The design of this review meant it was not appropriate or possible to involve patients or the public in the design, or conduct, or reporting, or dissemination plans of our research.

## Results

### Study selection

**Figure 1:**
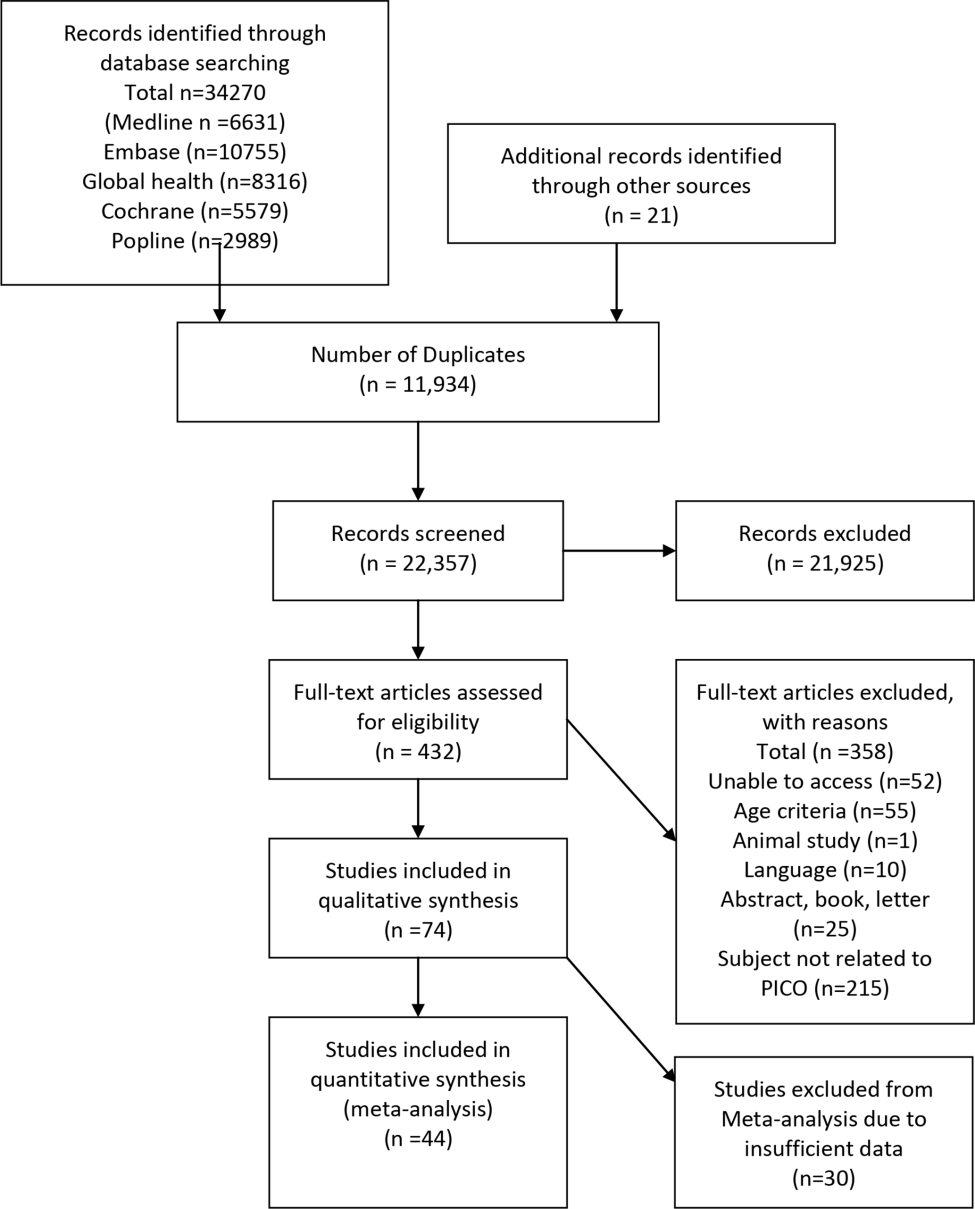
PRISMA flow diagram. PICO, Population, Intervention, Comparison, Outcome; PRISMA, Preferred Reporting Items for Systematic reviews and Meta-Analyses.

The study flow chart in figure 1 summarises our process of identifying studies. The final search of Embase, Global health, Popline and Cochrane databases conducted in March 2020 identified 34 270 studies, including both peer-reviewed studies and grey literature. In addition, 21 studies were found from other sources. After removing duplicates, 22 357 studies remained. Initial screening excluded 21 925 studies as they were unrelated to our review questions. Full texts of the 432 remaining studies were reviewed in detail to assess eligibility. At this stage, a further 358 studies were discarded as they did not meet the inclusion criteria, mostly because there was no mention of sex or gender in relation to undernutrition. Seventy-four studies were therefore included in the qualitative synthesis. Finally, we reviewed the 74 studies for inclusion in the meta-analysis and excluded 30 on the basis of insufficiently disaggregated data (which prevented the calculation of ORs). Thus, 44 studies were included in the meta-analysis.

### Study characteristics

Table 1 shows the characteristics of each of the studies included in the review. The studies selected for the review varied widely in terms of aims and study design. Many were observational, assessing prevalence of undernutrition and related risk factors and many included secondary data analysis. The outcomes, both primary and secondary, also varied widely. The studies took place in more than 30 countries (some covered multiple countries). The studies were spread across Central Africa (3/74) East Africa (33/74), East Asia (1/74), North Africa (1/74), Oceania (1/74), South America (2/74), South Asia (10/74), South East Asia (9/74), South West Pacific (1/74), West Africa (8/74) and multiple countries (5/74).

**Table 1:** Study characteristics

Study	Study design	Country	Region	Sample size	Boy/girl difference wasting	Boy/girl difference stunting	Boy/girl difference underweight	Reasons for differences discussed
Kismul H, *et al*.^36^	Cross-sectional	DRC	Central Africa	8994	NA	More boys	NA	No
Sakisaka K, *et al*.^37^	Cross-sectional	Nicaragua	Central Africa	755	NA	More girls	More girls	Yes
Vonaesch P, *et al*.^38^	Cross-sectional	CAR	Central Africa	414	NA	More boys	NA	Other results cited
Abdulahi A. *et al.* 39	Systematic review and meta-analysis	Ethiopia	East Africa	39 585	Not reported	More boys	Not reported	No
Abera L, Dejene T, Laelago T. 40	Cross-sectional	Ethiopia	East Africa	398	Not reported	NA	More boys	Other results cited
Abraham D, Elifaged H, Berhanu E.^41^	Cross-sectional	Ethiopia	East Africa	50	More boys	More boys	More boys	Yes
Altare C. *et al*. 42	Cross-sectional	Tanzania	East Africa	3264	NA	More boys	NA	Other results cited
Asfaw, M. *et al.* 43	Cross-sectional	Ethiopia	East Africa	796	More boys	More boys	More boys	Yes
Bukusuba J, Kaaya AN, Atukwase A. 44	Case–control	Uganda	East Africa	168	NA	More boys	NA	Yes
Chirande, L. *et al.* 45	Cross-sectional	Tanzania	East Africa	7324	NA	More boys	NA	Yes
Chirwa EW, Ngalawa HPE.^46^	Cross-sectional	Malawi	East Africa	13 858	More boys	More boys	More boys	Yes
Condo, J. *et al*. 47	Longitudinal	Rwanda	East Africa	480	More boys	More boys	More boys	Yes
Cruz, L. *et al*. 48	Case–control	Mozambique	East Africa	282	NA	More boys	NA	Yes
Tosheno D, Mehretie Adinew Y, Thangavel T, et al. 49	Cross-sectional	Ethiopia	East Africa	642	NA	NA	More boys	Yes
Eskezyiaw A, Tefera C. 50	Cross-sectional	Ethiopia	East Africa	562	NA	More boys	NA	No
Espo M, *et al*. 51	Cohort	Malawi	East Africa	613	NA	More boys	NA	No
Ettyang GA, Sawe CJ.^52^	Cross-sectional	Kenya and Cambodia	East Africa	10 163	NA	More boys	NA	Yes
Fentahun N, Belachew T, Lachat C. 53	Longitudinal	Ethiopia	East Africa	1927	More girls	More girls	More girls	Yes
Geresomo N, *et al*. 54	Cross-sectional	Malawi	East Africa	306	NA	More boys	NA	Yes
Gewa CA, Yandell N. 55	Cross-sectional	Kenya	East Africa	3793	More boys	More boys	More boys	Yes
Medhin G, *et al*^56^	Longitudinal	Ethiopia	East Africa	1799	NA	More boys	More boys	Yes
Habtom K, *et al*. 57	Cross-sectional	Ethiopia	East Africa	593	More boys	NA	More boys	No
Haile D, *et al*. 58	Cross-sectional	Ethiopia	East Africa	9893	NA	More boys	NA	Yes
Kinyoki DK, *et al*. 59	Cross-sectional	Somalia	East Africa	73 778	More boys	More boys	More boys	No
Masibo PK, Makoka D. 60	Cross-sectional	Kenya	East Africa	19 021	NA	More boys	More boys	No
Matanda DJ, Mittelmark MB, Kigaru DMD.^61^	Cross-sectional	Kenya	East Africa	11 938	More boys	More boys	NA	Yes
Mgongo M, *et al*. 62	Cross-sectional	Tanzania	East Africa	1870	More boys	More boys	More boys	Other results cited
Moges B, *et al*. 63	Cross-sectional	Ethiopia	East Africa	734	NA	More boys	NA	No
Ndemwa M, *et al*. 64	Cross-sectional	Kenya	East Africa	380	NA	More boys	More boys	Yes
Ndiku M, *et al*. 65	Cross-sectional	Kenya	East Africa	629	More girls	More girls	More girls	Yes
Ntenda PAM, Chuang YC. 66	Cross-sectional	Malawi	East Africa	6384	More boys	More boys	More boys	Yes
Olwedo MA, *et al*. 67	Cross-sectional	Uganda	East Africa	672	More boys	More boys	NA	Yes
Rakotomanana H, *et al*. 68	Cross-sectional	Madagascar	East Africa	4774	NA	More boys	NA	Yes
Tadesse AW, *et al*. 69	Cross-sectional	Ethiopia	East Africa	622	NA	More boys	NA	Yes
Yisak H, Gobena T, Mesfin F. 70	Cross-sectional	Ethiopia	East Africa	791	More boys	More boys	More boys	No
Yourkavitch J. 71	Cross-sectional	Rwanda	East Africa	1572	More boys	More boys	More boys	Yes
Jiang Y, *et al*.^72^	Cross-sectional	China	East Asia	1115	NA	More boys	NA	Other results cited
Díez-Navarro A, *et al*. 73	Cross-sectional	Multiple (Africa, Latin America, Asia)	Multiple	367 258	More boys	More boys	More boys	Yes
Khara T, *et al*. 28	Cross-sectional	Multiple (global)	Multiple	570 930	More boys	More boys	NA	Yes
Keino S, *et al*.^74^	Systematic review	Multiple (sub-Saharan Africa)	Multiple	195 559	NA	More boys	NA	No
Myatt M, *et al*.^29^	Meta-analysis	Multiple (global)	Multiple	1 796 991	More boys reported with concurrent wasting and stunting	NA		Yes
Wamani H, *et al*.^21^	Cross-sectional	Multiple (sub-Saharan Africa)	Multiple	64 000	NA	More boys	NA	Yes
El-Taguri A, *et al*. 75	Cross-sectional	Libya	North Africa	4498	NA	More boys	NA	No
Choy, C. *et al*. 76	Cross-sectional	Samoa	Oceania	305	NA	More boys	NA	Yes
Castro B.A. *et al*. 77	Cross-sectional	Columbia	South America	2967 under 5’s	NA	More girls	NA	Yes
Correia, L. *et al*. 78	Cross-sectional	Brazil	South America	6046	More girls	More girls	NA	No
Aguayo VM, Badgaiyan N, Paintal K. 79	Cross-sectional	Bhutan	South Asia	2085	NA	More boys	NA	Other results cited
Aguayo VM, Badgaiyan N, Dzed L.^80^	Cross-sectional	Bhutan	South Asia	2028	More boys	NA	NA	No
Baig-Ansari, N. *et al*. 81	Cross-sectional	Pakistan	South Asia	399	NA	More girls	NA	Yes
Biswas S, Bose K.^82^	Cross-sectional	India	South Asia	161	More boys	More girls	More boys	Yes
Gupta A. 83	Cross-sectional	India	South Asia	440	NA	More girls	NA	Yes
Khan, A.T. *et al*. 84	Cross-sectional	Pakistan	South Asia	3964	More boys	More boys	More boys	No
Kumar D, *et al*. 85	Cross-sectional	India	South Asia	424	NA	NA	More girls	Yes
Sand A, *et al*. 86	Cross-sectional	Pakistan	South Asia	105	More boys	NA	NA	No
Shaikh S, *et al*.^87^	Cross-sectional	India	South Asia	245	More boys	More girls	More girls	Yes
Shashank KJ, Angadi MM. 88	Cross-sectional	India	South Asia	161	More boys	More girls	More boys	Yes
Adair LS, Guilkey DK. 89	Longitudinal	Philippines	South East Asia	3080	NA	More boys	NA	Yes
Ahmed A.M. *et al* 90	Cross-sectional	Bangladesh	South East Asia	8858	More boys	More boys	More boys	Other results cited
Choudhury, N. *et al*. 91	Surveillance	Bangladesh	South East Asia	10 291	More boys	More boys	More boys	Other results cited
Chowdhury, M.R. *et al*.^92^	Cross-sectional	Bangladesh	South East Asia	7568	No difference found	More girls	More girls	Other results cited
Dancer D, Rammohan A, Smith MD.^93^	Cross-sectional	Bangladesh	South East Asia	5172	More girls	More girls	NA	Yes
Islam MM, *et al*. 94	Cohort	Bangladesh	South East Asia	265	NA	More boys	NA	Yes
Khambalia A, *et al*. 95	Literature review	Malaysia	South East Asia	NA	NA	More boys	No	NA
Phengxay M, *et al*. 96	Cross-sectional	Laos	South East Asia	798	More girls	More boys	More boys	No
Ramli, A. *et al*. 97	Cross-sectional	Indonesia	South East Asia	2168	NA	More boys	NA	No
Olita'a D, *et al*. 98	Case–control	Papua New Guinea	South West Pacific	50	More girls	More girls	More girls	Yes
Akombi BJ, *et al*. 99	Cross-sectional	Nigeria	West Africa	24 529	NA	More boys	NA	Yes
Amugsi DA, Mittelmark MB, Lartey A.^100^	Cross-sectional	Ghana	West Africa	7235	More boys	More boys	More boys	No
Bork KA, Diallo A.^32^	Cohort	Senegal	West Africa	7319	More boys	More boys	NA	Yes
Darteh EK, Acquah E, Kumi-Kyereme A. 101	Cross-sectional	Ghana	West Africa	2379	NA	More boys	NA	No
Garenne M, *et al*. 102	Longitudinal	Senegal	West Africa	12 638	More boys	More boys	NA	Yes
Miah RW, Apanga PA, Abdul-Haq Z. 103	Cross-sectional	Ghana	West Africa	7550	More boys	More boys	More boys	Yes
Olusanya BO, Wirz SL, Renner JK.^104^	Cross-sectional	Nigeria	West Africa	5994	More boys	More boys	More boys	No
Poda GG, Hsu C, Chao JCJ. 105	Cross-sectional	Burkina Faso	West Africa	6337	More boys	More boys	More boys	Other results cited

CAR, Central African Republic; DRC, Democratic Republic of the Congo; NA, not available.

Where sample size was clearly stated, the included studies involved 3 361 736 participants. Distribution of boys and girls was not provided for all studies but, where they were, results showed a total of 1 489 586 (44.3%) girls and 1 531 859 (45.6%) boys. Inclusion criteria for age entailed a mix of studies covering all children aged 0–59 months with others focused on subsets of these children.

### Meta-analysis

**Figure 2:**
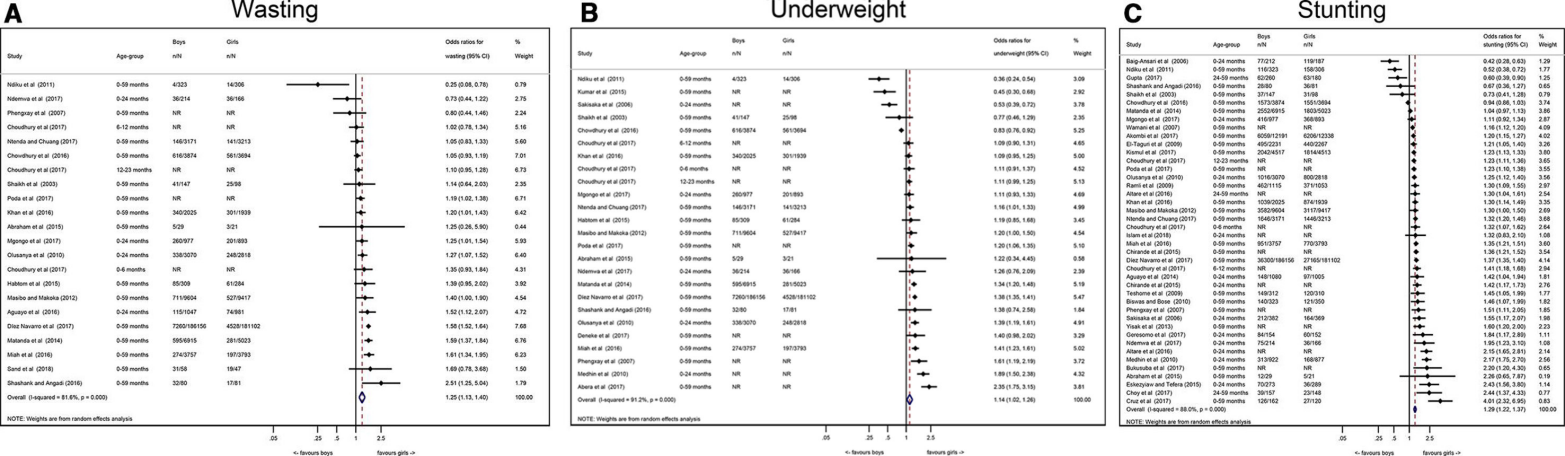
Forest plots showing the odds ratios for wasting, stunting and underweight in boys compared to girls.

We identified 74 studies that had measured undernutrition in the form of wasting, stunting and underweight and reviewed them for inclusion in the meta-analysis. Forty-four studies included extractable data, fully disaggregated by sex and were therefore eligible for inclusion, 41 of these were cross-sectional and 3 were longitudinal (in which case the most recent prevalence data were used). Results from the analysis are presented in the forest plots in figure 2.

### Pooled analysis by outcome

Twenty studies were included in the pooled analysis of wasting. In 17 of the 20 studies, wasting was more prevalent in boys than girls, with evidence of a difference in 11/17 of the studies. In the remaining three studies, wasting was more prevalent in girls than boys, with a significant difference in 1/3 of the studies. The pooled results of individual studies for wasting showed that boys had 26% higher odds of being wasted than girls (pooled OR 1.26, 95% CI 1.13 to 1.40, p<0.001).

Thirty-eight studies were included in the pooled analysis of stunting. In 32 of the 38 studies, stunting was more prevalent in boys than girls, with evidence of a difference in 28/32 of the studies. In the remaining six studies, stunting was more prevalent in girls than boys, with a significant difference in 3/6 of the studies. The pooled results for stunting showed that boys had 29% higher odds of being stunted than girls (pooled OR 1.29 95% CI 1.22 to 1.37, p<0.001).

Twenty-three studies were included in the pooled analysis of underweight. In 18 of the 23 studies, underweight was more prevalent in boys than in girls, with evidence of a difference in 10/18 of the studies. In the remaining five studies, girls were more likely to be underweight than boys, with a significant difference in 4/5 of the studies. The pooled results for underweight showed that boys had 14% higher odds of being underweight than girls (OR 1.14, 95% CI 1.02 to 1.26, p<0.001).

### Pooled analysis by region

When organised by region, the odds of boys being malnourished were nearly always higher than for girls for wasting, stunting and underweight. For wasting, the odds were higher for boys than for girls in all regions. For stunting, the odds were higher for boys than for girls in all regions except South Asia (pooled OR 0.88, 95% CI 0.62 to 1.26, p=0.492), where there was no difference by sex. For underweight, the odds were higher for boys than for girls in all regions except Central America (OR 0.53, 95% CI 0.40 to 0.72, p<0.001), although this finding was from a single study, and South Asia (pooled OR 0.84, 95% CI 0.52 to 1.35, p=0.475). Results from the analysis are presented in table 2.

**Table 2:** Odds of boys being undernourished compared with girls by regions and age groups

Region/age groups	No. of studies of wasting	Pooled OR (95% CI)	P value	No. of studies of stunting	Pooled OR (95% CI)	P value	No. of studies of underweight	Pooled OR (95% CI)	P value
Africa									
East	8	1.18 (0.95 to 1.47)	0.126	17	1.50 (1.29 to 1.72)	<0.001	11	1.24 (1.02 to 1.50)	<0.0034
West	3	1.34 (1.12 to 1.59)	0.001	4	1.24 (1.18 to 1.30)	<0.001	3	1.32 (1.19 to 1.47)	<0.001
Central				1	1.23 (1.13 to 1.33)	<0.001			
North				1	1.21 (1.05 to 1.40)	0.009			
Oceania				1	2.44 (1.37 to 4.33)	0.002			
Asia									
South	5	1.39 (1.12 to 1.72)	0.003	7	0.88 (0.62 to 1.26)	0.492	4	0.84 (0.52 to 1.35)	0.475
South East	3	1.08 (0.99 to 1.17)	0.092	5	1.25 (1.08 to 1.45)	0.003	3	1.09 (0.91 to 1.32)	0.350
Central America				1	1.56 (1.17 to 2.07)	0.003	1	0.53 (0.40 to 0.72)	<0.001
Multiple studies	1	1.58 (1.52 to 1.64)	<0.001	2	1.26 (1.07 to 1.49)	0.006	1	1.38 (1.35 to 1.41)	<0.001
Age group (months)									
0–24	5	1.19 (1.06 to 1.34)	0.004	12	1.46 (1.20 to 1.79)	<0.001	5	1.15 (0.80 to 1.65)	0.445
24–59				3	1.21 (0.63 to 2.33)	0.572			
0–59	15	1.30 (1.13 to 1.48)	<0.001	24	1.24 (1.16 to 1.32)	<0.001	17	1.13 (0.99 to 1.29)	0.066

### Pooled analysis by age

When organised by age groups, the odds of boys being wasted, stunted or underweight were higher in all age categories for boys than for girls. Results from the analysis are presented in table 2.

We repeated the analysis for relative risk and found the results were consistent with results for ORs. There was strong evidence of between-study heterogeneity of effect, (wasting I^2^=81.6%, p<0.001, stunting I^2^=88.0%, p<0.001, underweight I^2^=91.3%, p<0.001) which meta-regression by age group and region did not explain.

### Risk of bias within studies

The quality assessment can be seen in table 3. All studies presented appeared to be of acceptable quality. It is worth noting however that a number of studies were excluded prior to this process due to the absence of suitable data. The main differences in quality were in the domain which assessed whether sex differences were acknowledged and explored (see Qualitative synthesis section).

**Table 3:** Risk of bias assessment

Study	Data source	1	2	3	4	5	6	7	8	9
Abera L t al Dejene T, Laelago T.^40^	Community-based cross-sectional study	⬤	⬤	⬤	⬤	⬤	⬤	⬤	Other results cited	⬤
Abraham D, Elifaged H, Berhanu E.^41^	Community-based cross-sectional study	⬤	⬤	⬤	⬤	◐	⬤	⬤	⬤	◯
Aguayo VM, Badgaiyan N, Paintal K.^79^	BMIS—customised version of DHS	⬤	⬤	⬤	⬤	⬤	⬤	⬤	Other results cited	◯
Aguayo VM, Badgaiyan N, Dzed L.^80^	BMIS—customised version of DHS	⬤	⬤	⬤	⬤	⬤	⬤	⬤	◯	◯
Akombi BJ, *et al*.^99^	DHS 2013	⬤	⬤	⬤	⬤	⬤	⬤	⬤	⬤	⬤
Altare C. *et al*.^42^	Cross-sectional study	⬤	⬤	⬤	⬤	⬤	⬤	⬤	Other results cited	⬤
Baig-Ansari, N. *et al*.^81^	Cross-sectional survey	⬤	⬤	⬤	⬤	⬤	⬤	⬤	⬤	⬤
Biswas S, Bose K.^82^	Cross-sectional study	⬤	⬤	⬤	⬤	⬤	⬤	⬤	⬤	◯
Bukusuba J, Kaaya AN, Atukwase A.^44^	Case–control study	⬤	⬤	⬤	⬤	◯	⬤	⬤	⬤	⬤
Chirande, L. *et al*^45^	DHS 2010	⬤	⬤	⬤	⬤	◯	⬤	⬤	⬤	◯
Choudhury, N. *et al*.^91^	Surveillance data	⬤	⬤	⬤	⬤	◯	⬤	◐	Other results cited	⬤
Chowdhury, M.R. *et al*.^92^	DHS 2011	⬤	⬤	⬤	⬤	⬤	⬤	⬤	Other results cited	⬤
Choy, C. *et al*.^76^	Community-based cross-sectional study	⬤	⬤	⬤	⬤	⬤	⬤	⬤	⬤	⬤
Cruz, L. *et al*.^48^	Case–control study	⬤	⬤	⬤	⬤	⬤	⬤	⬤	⬤	⬤
Tosheno D, Mehretie Adinew Y, Thangavel T, *et al*^49^	Community-based cross-sectional study	⬤	⬤	⬤	⬤	⬤	⬤	⬤	⬤	◯
Díez-Navarro A, *et al*. 73	NGO intervention data	⬤	⬤	⬤	⬤	⬤	⬤	⬤	⬤	⬤
El-Taguri A, *et al*.^75^	Cross-sectional study	⬤	⬤	⬤	⬤	◐	⬤	⬤	◯	⬤
Eskezyiaw A, Tefera C.^50^	Community-based cross-sectional study	⬤	⬤	⬤	⬤	⬤	⬤	⬤	◯	⬤
Geresomo N, *et al*.^54^	Cross-sectional study	⬤	⬤	⬤	⬤	⬤	⬤	⬤	⬤	◯
Gupta A.^83^	Cross-sectional study	⬤	⬤	⬤	⬤	⬤	⬤	⬤	⬤	No
Habtom K, *et al*.^57^	Community-based cross-sectional study	⬤	⬤	⬤	⬤	⬤	⬤	⬤	◯	◐
Islam MM, *et al*.^94^	Longitudinal	⬤	⬤	⬤	⬤	⬤	⬤	⬤	⬤	⬤
Khan, A.T. *et al*.^84^	Cross-sectional study	⬤	⬤	⬤	⬤	⬤	⬤	⬤	◯	◯
Kismul H, *et al*.^36^	DHS 2013–2014	⬤	⬤	⬤	⬤	⬤	⬤	⬤	◯	◯
Kumar D, *et al*.^85^	Community-based cross-sectional study	⬤	⬤	⬤	⬤	⬤	◐	⬤	◯	◯
Masibo PK, Makoka D.^60^	DHS data—multiple years	⬤	⬤	⬤	⬤	⬤	⬤	⬤	◯	◯
Matanda DJ, Mittelmark MB, Kigaru DMD.^61^	DHS data—multiple years	⬤	⬤	⬤	⬤	⬤	⬤	⬤	⬤	⬤
Medhin G, *et al*.^56^	Surveillance data	⬤	⬤	⬤	⬤	⬤	⬤	⬤	⬤	⬤
Mgongo M, *et al*.^62^	Cross-sectional study	⬤	⬤	⬤	⬤	⬤	⬤	⬤	Other results cited	⬤
Miah RW, Apanga PA, Abdul-Haq Z. 103	Multiple indicator cluster survey data	⬤	⬤	⬤	⬤	⬤	⬤	⬤	Other results cited	⬤
Ndemwa M, *et al*.^64^	Cross-sectional study	⬤	⬤	⬤	⬤	⬤	⬤	⬤	Other results cited	◯
Ndiku M, *et al*.^65^	Cross-sectional study	⬤	⬤	⬤	⬤	⬤	⬤	⬤	⬤	◯
Ntenda PAM, Chuang YC.^66^	DHS multiple years	⬤	⬤	⬤	⬤	⬤	⬤	⬤	⬤	⬤
Olusanya BO, Wirz SL, Renner JK.^104^	Cross-sectional study	⬤	⬤	⬤	⬤	⬤	⬤	⬤	◯	⬤
Phengxay M, *et al*.^96^	Cross-sectional study	⬤	⬤	⬤	⬤	⬤	⬤	⬤	◯	◯
Poda GG, Hsu C, Chao JCJ.^105^	DHS 2010	⬤	⬤	⬤	⬤	⬤	⬤	⬤	Other results cited	⬤
Ramli, A. *et al*.^97^	Cross-sectional study	⬤	⬤	⬤	⬤	⬤	⬤	⬤	⬤	⬤
Sakisaka K, *et al*. 37	Cross-sectional study	⬤	⬤	⬤	⬤	⬤	⬤	⬤	◯	◯
Sand A, *et al*. 86	Cross-sectional study	⬤	⬤	⬤	⬤	⬤	⬤	⬤	◯	◯
Shaikh S, *et al*.^87^	Cross-sectional study	⬤	⬤	⬤	⬤	⬤	⬤	⬤	◯	◯
Shashank KJ, Angadi MM.^88^	Cross-sectional study	⬤	⬤	◯	⬤	◐	⬤	⬤	⬤	◐
Tadesse AW, *et al*.^69^	Cross-sectional study	⬤	⬤	⬤	⬤	⬤	⬤	⬤	⬤	◐
Wamani H, *et al*.^21^	DHS multiple years and countries	⬤	⬤	⬤	⬤	⬤	⬤	⬤	⬤	⬤
Yisak H, Gobena T, Mesfin F.^70^	Community-based cross-sectional study	⬤	⬤	⬤	⬤	⬤	⬤	⬤	◯	⬤

1. Were aims and objectives clearly stated?, 2. Was the target/reference population clearly described (is it clear who research is about)?, 3. Was a sample size justification, power description, or variance and effect estimates provided?, 4. Were the risk factor and outcome variables measured appropriate to the aims of the study?, 5. Were methods of anthropometric measurement and growth reference charts used well described?, 6. Is it clear what was used to determine statistical significance and/or precision estimates (eg, p values, CIs)?, 7. Results—were basic data adequately described?, 8. Were the male/female differences or non-differences discussed?, 9. Were limitations of study discussed?

⬤=Yes, ◯=No, ◐=Partially.

BMIS, Bhutan's Multiple Indicator Survey; DHS, Demographic and Health Surveys; NGO, non-governmental organisation.

### Qualitative synthesis

Seventy-four studies reported on outcomes related to undernutrition—wasting, stunting and underweight. From this, 38/74 studies reported on wasting as an outcome with 31/38 (81%) reporting a higher prevalence of wasting in boys, 6/38 (16%) reporting a higher prevalence of wasting in girls, 1/38 (3%) reporting no difference in the prevalence of wasting between boys and girls. Sixty-seven of 74 studies reported on stunting as an outcome. Fifty-four of 67 (81%) reported a higher prevalence of stunting in boys and 13/67 (19%) reported higher prevalence of stunting in girls. Thirty-five of 74 studies reported on underweight as an outcome. Twenty-eight of 35 (80%) reported higher prevalence of being underweight in boys, 7/35 (20%) reported a higher prevalence of underweight in girls.

We reviewed the discussion sections of the reports to see if these findings were explicitly acknowledged and if explanations were offered. Forty-three of 74 (58%) of the studies discussed the findings, 10/74 (14%) studies cited articles with similar findings but did not speculate as to the causes of these differences and 21/74 (28%) of the studies did not discuss the findings related to sex differences at all.

Among those study reports that did offer explanations for sex differences, the reasons varied widely and were often conjectural. We coded explanations as either biological (6/43; 14%), social (21/43; 49%) or a combination of the two (16/43; 37%). Biological reasons varied from a simple statement of ‘biological differences’ to more detailed exploration of sex differences in the immune and endocrine system between boys and girls. Social reasons given varied widely and were almost entirely conjectural, with exceptions identified through regression analysis related to son preference and related to sibling order and sex. Other social reasons given were gender dynamics, preferential feeding practices for either boys or girls, infant and young child feeding practices such as early weaning for boys and children’s behaviours whereby girls might stay closer to the home and have more access to food being cooked, while boys play outside and in turn eat less while expending more energy.

## Discussion

This review offers a systematic look at sex differences over a wide geographical area. The studies included in the meta-analysis show that boys aged 0–59 months are much more likely to be wasted, stunted and underweight using anthropometric case definitions than girls. This indicates sex differences in susceptibility to undernutrition. The reasons currently provided for these differences vary and are often speculative rather than informed by direct evidence.

When stratified by region, the results also showed that boys are more likely to be wasted, stunted or underweight than girls. There were however some exceptions where ORs were reduced or reversed for boys with respect to undernutrition, in East Africa, Central America, South and South East Asia. The differences in Central America were based solely on one study, with a limited sample size and therefore need to be interpreted with caution. Our analysis potentially masks some of the complexities of regional variations in sex differences, particularly in South and South East Asia as many studies from these regions did not qualify for inclusion in the meta-analysis due to insufficient data. It is possible these differences might be under or overestimated. In reviewing the individual studies identified in the main search, results from this region are inconsistent and often conflicting compared with those coming from other regions of the world, such as Africa, which show a more consistent pattern of male disadvantage, a finding resonating with other studies.^5 21^ The inconsistencies in findings for parts of South and South East Asia, however, may be explained in part by well-described social preferences for men,^22^ and warrant further investigation. Such differences have also been described for under-5 mortality, with excess female child mortality for certain diseases, and according to socioeconomic status, birth order and family composition.^23–26^

These findings challenge commonly held assumptions within the nutrition community that girls are more likely to be affected by undernutrition. Recent studies focused on the relationship between wasting and stunting have also highlighted similar findings showing boys are more likely to be concurrently wasted and stunted than girls^4 27–29^ and have identified this as an unexpected finding.

We found that even where sex differences are reported, they are not always acknowledged or explored. Just over a quarter of studies (28%) did not provide any discussion on reported differences and 14% cited similar findings but did not consider causes. Where explanations for sex differences in the prevalence of undernutrition were offered, nearly half (49%) of the studies reviewed offered explanations related to social reasons or based on speculation or preconceived supposition rather than evidence. The search criteria used (which filtered articles to those which use terms related to sex or gender in the abstract) might have introduced some bias here with a potential overestimation of studies that report and explore the issue of sex differences.

When stratified by age, the meta-analysis also shows that boys are at higher risk across all age groups, though again, our analysis potentially masks some of the complexities in age as detailed analysis of different age groups was not possible. While the results for age show that boys are more likely to be stunted than girls, the ORs are lower in the older age group compared with the younger group. Limited data in the 24–59 month age category, especially for wasting and underweight, however mean results must be interpreted with caution. These tentative results might indicate any sex-specific risks differ at different ages: further study is warranted. Two studies exploring concurrent wasting and stunting^28 29^ found it to be a condition that affects children below 30 months more than it does older children, and found that sex ratios in undernourished children change with age, with a higher susceptibility for boys up to 30 months that then disappeared. Alongside other studies,^30^ they suggest that sex hormones, specifically testosterone, luteinising hormone and follicle-stimulating hormones might play a role in this. Selection effects might also contribute to this, whereby if boys are more likely to die than girls, the remaining pool of boys would represent healthy survivors.

Adair and Guilkey^31^ studied children in the Philippines and found men were more likely to become stunted in the first year of life (using the NCHS reference), but women were more likely than men to become stunted in the second year. They suggest differences in parental caregiving behaviours may partly account for this finding, but these were not measured in the study. Bork and Diallo^32^ also found evidence of interaction between age and sex in that the deficit in boys compared with that in girls increased between the first and second years of life, regardless of the indicator used. The differences in height status were however sensitive to the growth reference chosen; they were greater when assessed using the 2006 WHO growth standards than when using the NCHS growth reference.

Sex differences in undernutrition may vary not only by geographical area, but also over time. When diseases causing undernutrition known to be more severe among girls, such as measles, whooping cough and tuberculosis, disappear because of vaccination, lower transmission and better feeding, the disadvantage of boys might increase. Conversely, if efficient nutrition programmes are conducted, the disadvantage of boys might be reduced over the years.

Interpretation of these findings into implications for practice and policy is limited at this stage but does warrant consideration and some degree of change. As a minimum, the systematic collection and reporting of disaggregated data by age and sex should be included in the design of programmes and assessments in all settings. Where differences are observed, particularly in programme admissions, these should be interpreted in light of sex differences in population burden in order to draw conclusions as to whether programmes are proving equally accessible to boys and girls, and then the potential causes of these differences should be considered and/or investigated. At present, boys’ vulnerability to undernutrition is rarely a consideration in the design of nutrition programming, nor the formulation of policy. Moreover, some high-level international nutrition policies explicitly focus on the vulnerability of women and girls (eg, The Scaling Up Nutrition Movement Road Map for 2016–2020 Khara *et al*^28^). Similarly, the recent Inter-Agency Standing Committee guidance on gender in humanitarian action^33^ recognises the inequity in food intake that may be faced by women and girls in crises but makes no reference to higher levels of undernutrition among boys. The absence of any reflection on gender, or the misuse of the term to highlight solely the health of women and girls, is likely to unintentionally reinforce inequalities in health.^7^ In the Nutrition for Growth 2020 summit (https://nutritionforgrowth.org/) and beyond, a major focus will be on inequities in undernutrition and how they affect different groups in different locations. The emerging findings from this review have significance in ensuring consideration of these sex differences through an equity lens.

### Strengths and limitations

One of the strengths of this study lies in the systematic approach that was chosen and its primary objective to review sex differences in undernutrition over a wide geographical area. However, there are areas where bias has potentially been introduced.

First, screening for studies to be included in this study was conducted by only one of the authors. While we employed systems to ensure contentious articles were discussed among two or more authors, we recognise that not using double screening is a limitation.^34^

Second, the search strategy looking for explicit mention of sex or gender in the abstract might have biased towards studies that reported on sex and gender in the abstract, or towards studies that found a significant difference, and therefore sex differences might be under-reported or over-reported in this study. Likewise, the search may have limited the analysis as there are potentially missed studies which include sex as a variable in analysis but without focusing on mention of sex in the study abstracts. Similarly, there may be a degree of publication bias whereby sex differences are simply not considered or reported.

The search criteria also encompassed a large number of studies with differing objectives meaning a limited degree of homogeneity. Few studies directly assessed the true relation between sex and undernutrition. This analysis is therefore potentially biased by healthy survivors—those children that have survived to be included in studies. We do not believe however that our results would be significantly different considering the evidence presented on male vulnerability. We also recognise the possibility of an overlap in data used from sources such as Demographic and Health Surveys (DHS). By comparing the dates and locations of included studies, we have not been able to establish any overlap. Unidentified overlap, if it occurs, is therefore likely to be minimal in our review and unlikely to affect overall conclusions. Where multiple studies are available from the same country, we have established these to be from different regions and times, therefore their inclusion as independent studies is justified. We hope that our review will stimulate future work to explore not just intercountry differences but also intracountry/regional differences as this would help understand biological versus social reasons for any difference in male/female risks.

While this analysis included some secondary DHS data, the subject in question could benefit from a systematic analysis of DHS, Multiple Indicator Cluster Surveys and or nutrition survey data. Though it is not believed that the outcome of the ORs of sex differences would be different, further analysis might help improve understanding of some of the complexities of age, context, dual burdens of undernutrition and sex differences and the implications for programmers. This might also help towards explaining some of the between-study heterogeneity that we identified but were unable to explain with our analysis.

The rigour of findings of the analysis is limited in relation to age as the grouping and degree of available data potentially masks some of the differences at various stages of the lifecycle, similarly geographical differences might be biased towards studies included through the search.

The absence of data on other anthropometric measures, such as Mid-Upper Arm Circumference (MUAC), is also a potential limitation. In considering the implications of the differences highlighted here, in addition to biological and social explanations, it is necessary to consider how we measure and define undernutrition and whether sex differences are an artefact of the indices in use. The WHO growth standards describe the physiological growth within optimal environmental conditions and are separated by sex. These reference data from healthy well-nourished populations resolve sex differences to zero by expressing data as z-scores calculated using the appropriate male and female subset of the reference population. However, it is unclear if we would expect sex differences in undernutrition expressed in this way to be zero, when the distribution of weight and height in both sexes has been shifted away from the healthy reference range. Likewise, it is unclear if the loss of the same amount of body weight in a girl or boy would have the same physiological effect. If boys cope worse than girls when exposed to food shortages or disease and infection, this potentially highlights increased vulnerability over and above what is already accounted for by the standards.

In comparison, MUAC cut-offs are unadjusted and do not differentiate by sex or age (between 6 months and 5 years). This absence of adjustment may lead to a preferential inclusion of girls in programmes compared with what would be obtained if sex-specific standards were used as girls tend to have lower MUACs than boys. Though it has been shown to be a good predictor of mortality, sex differences in using MUAC to define undernutrition have not been widely studied.

Finally, the number of studies identified in the overall search that qualified for the meta-analysis was low. This was mainly due to a lack of presentation of disaggregated data. A recent Lancet series on gender equality, norms and health, highlighted the need for accurate disaggregated data.^35^

### Implications for future research

This study is a step towards better understanding of sex differences in undernutrition and highlights the need to consider potential implications for policy and practice. Future research should aim to unpack the complexities related to age, biological and social risks (including gender norms) and context. In particular, we note that current papers are conjectural about reasons for observed differences. Any hypotheses should be more directly tested in future studies to further our understanding of sex differences in the context of undernutrition and subregional variations in order to determine the implications of these differences for programme staff and policymakers.

Future research will focus on a more detailed analysis of factors affecting outcomes for boys and girls such as epidemiological, demographic and social differences, explore the consequences of sex, age, and behavioural differences in nutritional outcomes and mortality. The impact of using differing anthropometric measurement and indices should also be explored to better understand how differing methods detect the most vulnerable children and explore how substantial sex differences are.

## Conclusion

This review demonstrates that undernutrition defined by anthropometric case definitions is usually higher among boys than girls. While further research is needed to understand the policy and programming implications of these differences, lessons can already be drawn from this research. We call on nutrition actors to improve data collection in programmes, surveys and research through the full disaggregation and analysis of sex and age in order to identify which children are most vulnerable in specific contexts, and to allow comparison of programme data with population-level burdens. It is important to understand that the message of this study is not that boys should be prioritised over girls, rather it seeks to support all at-risk children, through improved understanding of sex differences in undernutrition. Ultimately, we believe all children under 5 years and their caregivers should be seen as a high priority group for targeted nutrition interventions, and resources and interventions should be targeted according to need.
